# Two *Listeria monocytogenes* outbreaks in a cancer centre: onsite food premises and their potential health risk to patients

**DOI:** 10.1186/s12889-023-16371-7

**Published:** 2023-07-28

**Authors:** J. Leigh Hobbs, Christina Lee, Brian Thompson, Adrienne Andrew, Christine Navarro, Vinita Dubey, Anne Maki, Adrienne Kong, Meghan Griffin, Kelvin Chau, Allana M. Murphy, Marina Lombos, Anna L. Majury, Monica Gerrie, Erin Szidonya, Jackson Chung, Omar Ozaldin, Toral Patel, Nicholas Brandon, Bryna Warshawsky

**Affiliations:** 1 Public Health Ontario, 661 University Avenue, Suite 1701, Toronto, ON M5G 1M1 Canada; 2 Toronto Public Health, 277 Victoria St, Toronto, ON M5B 1W2 Canada; 3 Canadian Food Inspection Agency, Office of Food Safety and Recall, 1400 Merivale Road, Ottawa, ON K1A 0Y9 Canada; 4 Public Health Agency of Canada National Microbiology Laboratory, 1015 Arlington St, Winnipeg, MN R3E 3P6 Canada; 5 University of Toronto, 27 King’s College Cir, Toronto, ON M5S 1A1 Canada; 6 Western University, 1151 Richmond St, London, ON N6A 3K7 Canada

**Keywords:** Listeriosis, *Listeria monocytogenes*, Deli meat, Outbreak, Food safety, Foodborne illness, Cancer, Immuno-compromising condition, Hospital, Health care

## Abstract

**Background:**

This report describes two *L. monocytogenes* outbreak investigations that occurred in March and September of 2018 and that linked illness to a food premises located in an Ontario cancer centre. The cancer centre serves patients from across the province.

**Methods:**

In Ontario, local public health agencies follow up with all reported laboratory-confirmed cases of listeriosis to identify possible sources of disease acquisition and to carry out investigations, including at suspected food premises. The Canadian Food Inspection Agency (CFIA) is notified of any *Listeria*-positive food product collected in relation to a case. The CFIA traces *Listeria*-positive product through the food distribution system to identify the contamination source and ensure the implicated manufacturing facility implements corrective measures.

**Results:**

Outbreaks one and two each involved three outbreak-confirmed listeriosis cases. All six cases were considered genetically related by whole genome sequencing (WGS). In both outbreaks, outbreak-confirmed cases reported consuming meals at a food premises located in a cancer centre (food premises A) before illness onset. Various open deli meat samples and, in outbreak two, environmental swabs (primarily from the meat slicer) collected from food premises A were genetically related to the outbreak-confirmed cases. Food premises A closed as a result of the investigations.

**Conclusions:**

When procuring on-site food premises, healthcare facilities and institutions serving individuals with immuno-compromising conditions should consider the potential health risk of foods available to their patient population.

## Background

Listeriosis is a bacterial infection caused by the pathogen *Listeria monocytogenes*. In healthy individuals, infection with *L. monocytogenes* is typically asymptomatic or a mild, self-limited illness such as febrile gastroenteritis. However, listeriosis in individuals with immuno-compromising conditions can result in severe or potentially fatal illness, including septicemia or meningitis. Pregnant women are at increased risk for *Listeria* infections which can result in stillbirth or miscarriage [1]. Disease surveillance data from Ontario, Canada, from 2011 to 2019 indicates approximately 62 individuals (0.40 cases per 100,000 population) are reported with listeriosis yearly [2]. Further, the proportion of reported cases with severe outcomes is high. Based on data from 2019, 75.3% of Ontario cases were reported as hospitalized and a fatal outcome was reported for 19.2% of cases [2].

*L. monocytogenes* is primarily acquired through consuming contaminated food. Internationally, outbreaks have been linked to ready-to-eat meats, soft cheese, and other dairy products [3–22]. Additional sources of *L. monocytogenes* outbreaks have also been identified, including whole apples, smoked fish, ready-to-eat salads, and cantaloupes [23–27]. Also, in 2015-16, an outbreak confined to Ontario, which resulted in 34 cases and 4 deaths, was attributed to pasteurized chocolate milk [28].

Listeriosis outbreaks affecting institutions, such as hospitals and long-term care homes, have also been described. In 2008, a large Canadian outbreak involving 57 laboratory-confirmed cases and 24 deaths, mainly from Ontario, was linked to ready-to-eat deli meats purchased primarily by institutions [29]. Similarly, in Denmark an outbreak was linked to spiced meat rolls served in hospitals and long-term care homes [30]. Hospital-acquired outbreaks have also been linked to soft cheese, diced celery, and pasteurized ice cream mix [31–34]. In particular, several hospital outbreaks in the United Kingdom were associated with pre-made sandwiches with mixed fillings [35–37]. The sandwiches were produced off-site in these outbreaks and contaminated at the production facilities.

This report aims to describe two *L. monocytogenes* outbreak investigations that linked illness to a food premises located in a cancer centre in Ontario (food premises A). The on-site food premises was open to the public and the in- and out-patient population seeking medical care at the cancer centre. The report will highlight issues raised from the investigations, including policy considerations for healthcare facilities and institutions, particularly those serving individuals with immuno-compromising conditions, about the type of food premises permitted on-site.

## Methods

### *Listeria* surveillance and outbreak investigations

Laboratories and health care providers in Ontario are required under provincial legislation to report laboratory-confirmed and clinical cases of designated diseases of public health significance, including listeriosis. Laboratory-confirmed cases of listeriosis are considered invasive as confirmation requires the isolation of *L. monocytogenes* from a normally sterile site (e.g., blood). Cases are reported through the Ontario Ministry of Health (MOH) integrated Public Health Information System (iPHIS), the reportable disease database for the province, and monitored provincially by Public Health Ontario (PHO) for surveillance and outbreak detection. When a multi-jurisdictional outbreak (i.e., an outbreak involving two or more local public health units) within Ontario is detected, PHO leads the investigation. Local public health officials routinely follow up with reported cases of listeriosis using a national standardized questionnaire to identify possible sources of disease acquisition and to conduct case management. Details collected during routine follow up include cases’ food histories and food premises at which the case dined four weeks prior to illness onset. Food premises reported by cases are typically investigated and, if warranted, samples of suspect food items consumed by the case and environmental swabs are collected. Food samples and environmental swabs are tested at the PHO Laboratory, Ontario’s public health reference laboratory.

The Canadian Food Inspection Agency (CFIA) is notified of any *Listeria*-positive food product collected by local public health officials in relation to a case of listeriosis. Following notification of a *Listeria*-positive food product, the CFIA conducts a food safety investigation. The CFIA traces back *Listeria*-positive food through the distribution system to manufacturing facilities, reviews records and processes at facilities, and conducts representative sampling and analysis of intact food units (i.e., closed samples) and environmental swabbing to determine a possible source and the extent of environmental contamination. CFIA also traces the implicated food product forward to determine if a product that poses a health risk to Canadians is available on the market. Risk mitigating action may be requested whereby the responsible regulated party conducts a recall and withdraws the affected product from the market. Following a recall, the CFIA verifies its effectiveness, requests that the firm identify the cause of the contamination, and ensures that corrective actions are implemented to prevent similar issues from occurring in the future.

### Outbreak case definitions

The first outbreak was declared in March 2018, and a confirmed case was defined as a resident or visitor to Ontario with laboratory confirmation of *L. monocytogenes* with the PulseNet Canada whole genome sequencing (WGS) cluster code 1803LMWGS-1ON and symptom onset on or after 1 January 2018. An outbreak-probable case was defined as a resident or visitor to Ontario with laboratory confirmation of *L. monocytogenes* with an epidemiological link to food premises A and symptom onset on or after 1 January 2018. For the second outbreak, which was declared in September 2018, a confirmed case was defined as a resident or visitor to Ontario with laboratory confirmation of *L. monocytogenes* with the PulseNet Canada WGS cluster code 1810LMWGS-1ON and symptom onset on or after 15 August 2018. An outbreak-probable case was defined as a resident or visitor to Ontario with laboratory confirmation of *L. monocytogenes* with an epidemiological link to food premises A and symptom onset on or after 15 August 2018.

### PHO laboratory testing methods

Clinical isolates of *L. monocytogenes* identified in hospital and private laboratories are submitted to the PHO Laboratory for molecular subtyping and surveillance purposes. Food samples and environmental swabs collected by public health unit officials in relation to a *L. monocytogenes* clinical case are also submitted to the PHO Laboratory, and testing is performed according to the Health Canada method MFHPB-30 [38]. During the outbreak investigations molecular subtyping by both pulsed field gel electrophoresis (PFGE) and WGS was performed in accordance with PulseNet Canada standard operating procedures; both methods were used as the PHO Laboratory was transitioning methods during this time. During the first outbreak, PFGE was performed for the clinical, food and environmental *L. monocytogenes* isolates at the PHO Laboratory and WGS was performed at the National Microbiology Laboratory (NML) in Winnipeg, Manitoba, Canada. PFGE and WGS for the second outbreak were both performed at the PHO Laboratory. Comparisons were generated using BioNumerics version 7.6 and nodes were calculated using UPGMA (Unweighted Pair Group Method with Arithmetic Mean), which is the standard analytical process for PulseNet Canada laboratories. Comparative analysis of WGS results from both outbreaks one and two were performed by the NML.

### CFIA laboratory testing methods

Food samples from retail and production facilities collected for testing by the CFIA were analyzed at CFIA laboratories according to Health Canada methods MFLP-28 and MFHPB-30, and environmental samples from production facilities were analyzed according to methods MFLP-15 and MFHPB-30 [38]. *L. monocytogenes* positive food samples were enumerated according to the Health Canada method MFLP-74 [38]. Molecular subtyping by both PFGE and WGS for the food and environmental *L. monocytogenes* isolates was performed at CFIA laboratories in accordance with PulseNet Canada standard operating procedures.

## Results

### Outbreak One

**Fig. 1:**
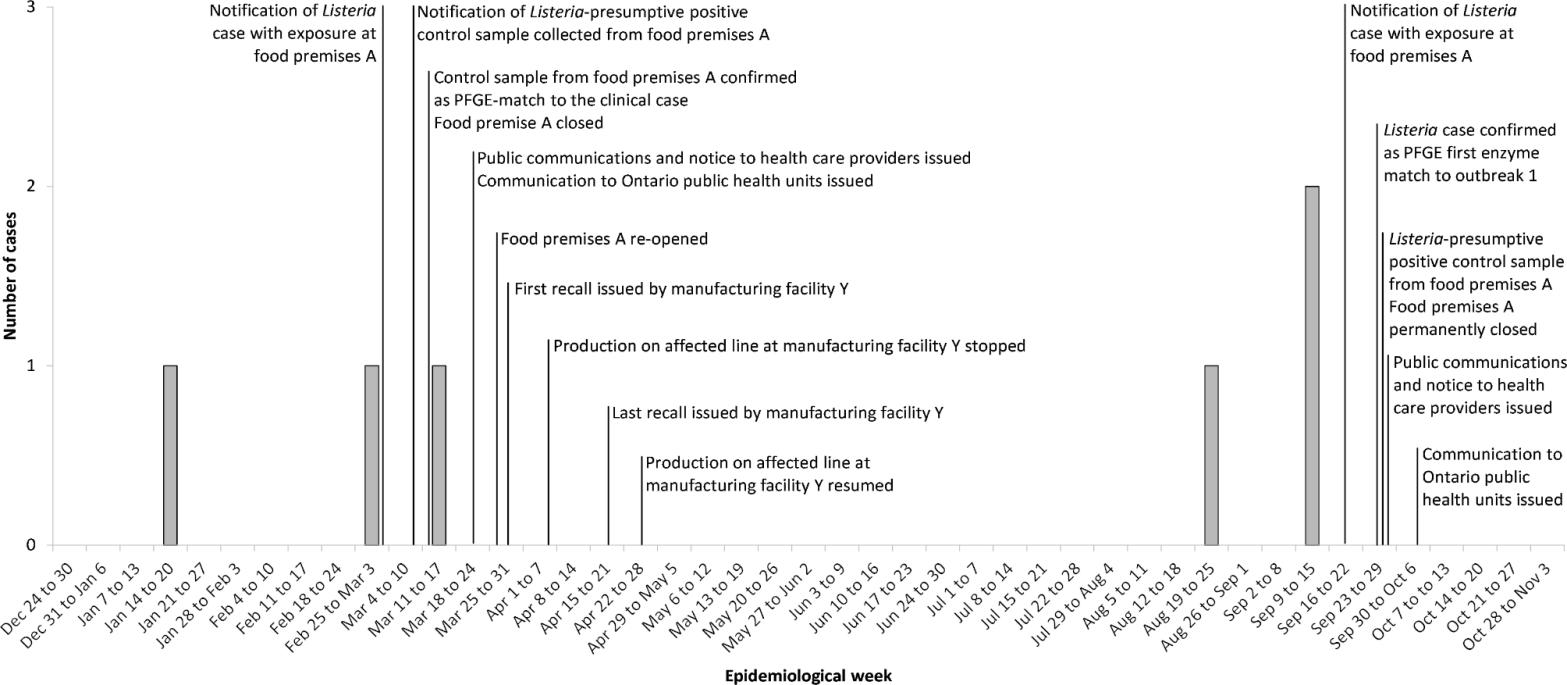
Timeline of key events related to two listeriosis outbreaks associated with a food premises located in a cancer center (food premises A), Ontario, 2018

In early March 2018, local public health officials received a report of a laboratory-confirmed case of *L. monocytogenes*. During routine case follow-up, it was determined that the case was receiving cancer treatment at an Ontario cancer centre and had consumed a meal at food premises A during their incubation period (i.e., the four weeks before illness onset). Food premises A was part of a chain of restaurants with locations throughout Ontario, one of which was in the cancer centre. As food premises reported by any confirmed listeriosis case during their incubation period are typically investigated, control samples of menu items consumed by the case were collected for testing. Seven days later, local public health officials were notified that the control sample of sliced corned beef collected from food premises A was presumptive positive for *Listeria* (Fig. 1). Four days following the presumptive positive finding, the open control sample of corned beef was confirmed to be a PFGE-match to the case and food premises A was closed on the same day. The public health unit in whose jurisdiction food premises A was located issued a media release to inform the public of the investigation; as well, targeted communications were sent to health care providers to assist with prompt diagnosis and treatment of cases. PHO also issued a communication to all public health units in Ontario requesting they share information about the outbreak with health care providers in their jurisdiction.

**Fig. 2:**
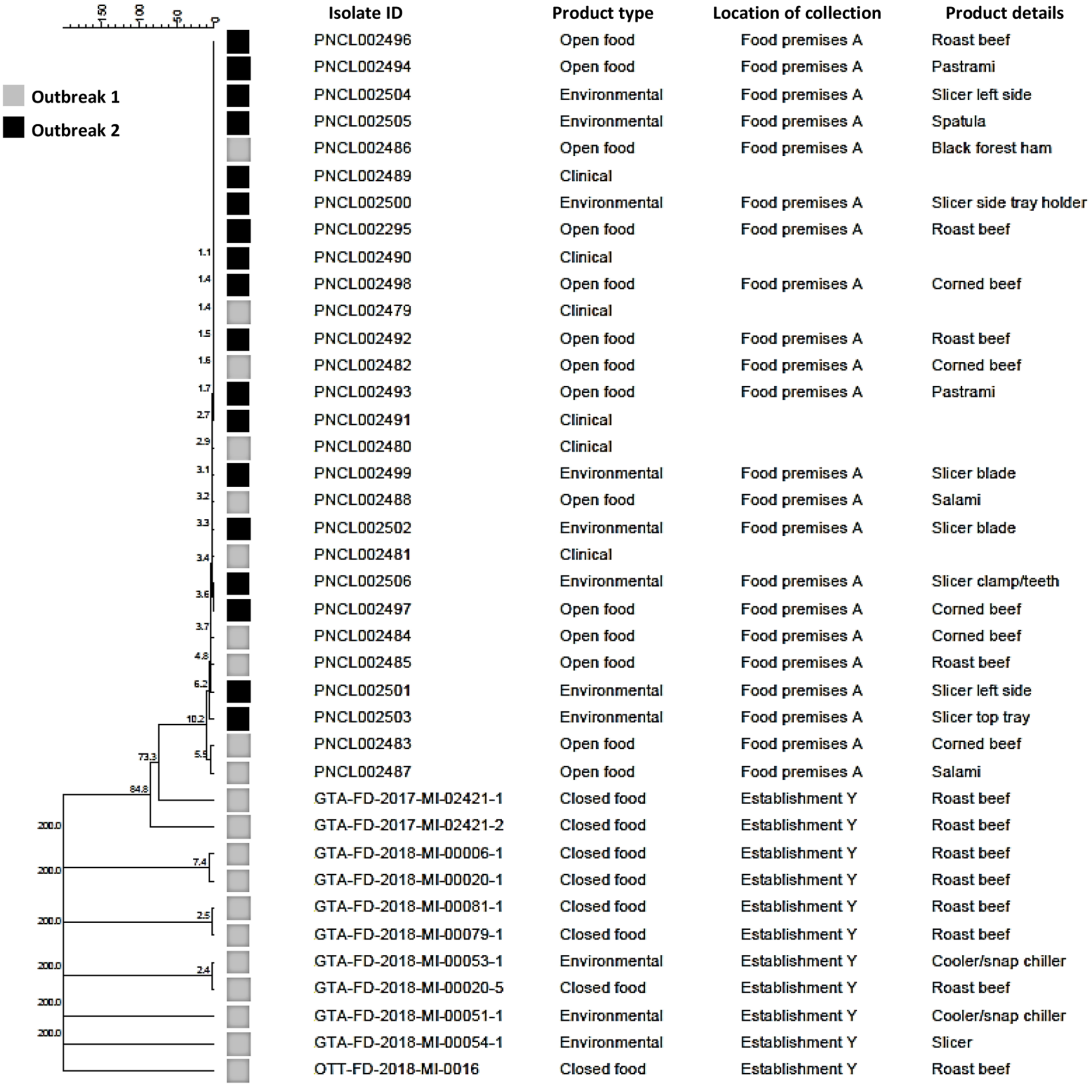
Whole genome multi locus sequence typing (wgMLST) analysis of 39 clinical, food, and environmental isolates from two listeriosis outbreaks associated with a food premises located in a cancer center (food premise A), Ontario, 2018

A total of four laboratory-confirmed (three outbreak-confirmed and one outbreak-probable) cases from three different public health units in southern Ontario were included in the investigation. The three outbreak-confirmed cases were considered genetically related by WGS (within 0 to 10 alleles; Table 1; Fig. 2) and symptom onset ranged from mid-January to mid-March 2018. All three outbreak-confirmed cases were cancer patients receiving treatment at the cancer centre and were hospitalized due to listeriosis, and no deaths were reported during the course of the investigation. All three outbreak-confirmed cases reported consuming sandwiches at food premises A during their incubation period. The outbreak-probable case was a cancer patient with a symptom onset in late March but was not considered genetically related to the outbreak-confirmed cases by WGS (over 200 alleles different). The proxy for the outbreak-probable case reported that the case consumed meals at food premises A, however, further details could not be confirmed.

**Table 1:** Laboratory results for outbreak-confirmed and probable clinical cases from the two listeriosis outbreaks associated with a food premises located in a cancer center (food premises A), Ontario, 2018

	Isolate ID	Outbreak classification	WGS strain	Outbreak strain
**Outbreak 1**	PNCL002479	Confirmed	Strain 1	Yes
	PNCL002480	Confirmed	Strain 1	Yes
	PNCL002481	Confirmed	Strain 1	Yes
	NA^*^	Probable	Strain 10	No
**Outbreak 2**	PNCL002489	Confirmed	Strain 1	Yes
	PNCL002490	Confirmed	Strain 1	Yes
	PNCL002491	Confirmed	Strain 1	Yes

^*^ The isolate for the outbreak-probable case was not included in the whole genome sequencing (WGS) analysis, therefore an isolate ID is not available

Local public health officials collected 14 open control samples of various types of deli meat that had been sliced on-site at food premises A and 42 environmental swabs from the food premises. A total of five different open deli meat samples (a sample of roast beef, salami, and black forest ham, and two samples of corned beef) tested positive for *L. monocytogenes* and all *L. monocytogenes* isolates were genetically related to the outbreak-confirmed cases by WGS (within 0 to 10 alleles) (Table 2). *L. monocytogenes* was not detected in any environmental swabs, including swabs from the slicer, however, the environmental swabs were taken from the slicer following supervised cleaning and sanitization. Despite this, the slicer was a suspect source of cross-contamination as all the positive open samples had been sliced on-site with the same slicer and it was determined that the slicer was not being routinely dismantled, cleaned, and sanitized as required per the manufacturer’s specifications.

**Table 2:** Laboratory results and product details for *L. monocytogenes*–positive food samples and environmental swabs collected from the two listeriosis outbreaks associated with a food premises located in a cancer center (food premises A), Ontario, 2018

	Isolate ID	Product details	Product type	Location of collection	WGS strain(s)	Outbreak strain
**Outbreak 1**	PNCL002482	Corned beef	Open food	Food premises A	Strain 1	Yes
	PNCL002483	Corned beef	Open food	Food premises A	Strain 1	Yes
	PNCL002484					
	PNCL002485	Roast beef	Open food	Food premises A	Strain 1	Yes
	PNCL002486	Black forest ham	Open food	Food premises A	Strain 1	Yes
	PNCL002487	Salami	Open food	Food premises A	Strain 1	Yes
	PNCL002488					
	GTA-FD-2017-MI-02421-1	Roast beef	Closed food	Manufacturing facility Y	Strain 2	No
	GTA-FD-2017-MI-02421-2				Strain 3	
	GTA-FD-2018-MI-00006-1	Roast beef	Closed food	Manufacturing facility Y	Strain 4	No
	GTA-FD-2018-MI-00020-1	Roast beef	Closed food	Manufacturing facility Y	Strain 4	No
	GTA-FD-2018-MI-00020-5				Strain 6	
	GTA-FD-2018-MI-00081-1	Roast beef	Closed food	Manufacturing facility Y	Strain 5	No
	OTT-FD-2018-MI-0016	Roast beef	Closed food	Manufacturing facility Y	Strain 9	No
	GTA-FD-2018-MI-00079-1	Roast beef	Closed food	Manufacturing facility Y	Strain 5	No
	GTA-FD-2018-MI-00054-1	Slicer	Environmental	Manufacturing facility Y	Strain 8	No
	GTA-FD-2018-MI-00051-1	Cooler/snap chiller	Environmental	Manufacturing facility Y	Strain 7	No
	GTA-FD-2018-MI-00053-1	Cooler/snap chiller	Environmental	Manufacturing facility Y	Strain 6	No
**Outbreak 2**	PNCL002492	Roast beef	Open food	Food premises A	Strain 1	Yes
	PNCL002493	Pastrami	Open food	Food premises A	Strain 1	Yes
	PNCL002494	Pastrami	Open food	Food premises A	Strain 1	Yes
	PNCL002295	Roast beef	Open food	Food premises A	Strain 1	Yes
	PNCL002496	Roast beef	Open food	Food premises A	Strain 1	Yes
	PNCL002497	Corned beef	Open food	Food premises A	Strain 1	Yes
	PNCL002498	Corned beef	Open food	Food premises A	Strain 1	Yes
	PNCL002499	Slicer blade	Environmental	Food premises A	Strain 1	Yes
	PNCL002500	Slicer side tray holder	Environmental	Food premises A	Strain 1	Yes
	PNCL002501	Slicer left side	Environmental	Food premises A	Strain 1	Yes
	PNCL002502	Slicer blade	Environmental	Food premises A	Strain 1	Yes
	PNCL002503	Slicer top tray	Environmental	Food premises A	Strain 1	Yes
	PNCL002504	Slicer left side	Environmental	Food premises A	Strain 1	Yes
	PNCL002505	Spatula	Environmental	Food premises A	Strain 1	Yes
	PNCL002506	Slicer clamp/teeth	Environmental	Food premises A	Strain 1	Yes

^*^ Only *L. monocytogenes*-positive results are shown. Other food and environmental samples were tested and *L. monocytogenes* was not detected

^†^ Isolates are considered genetically related if they differ by approximately ten alleles or less

^‡^ For each food sample and environmental swab, multiple isolates were tested. Multiple isolates from the same sample are shown if WGS results differed by approximately ten or more alleles. For example, two isolates from each of two closed roast beef samples collected from manufacturing facility Y are shown, as these isolates differed by more than ten alleles and thus are considered genetically unrelated (strains 2 and 3, and strains 4 and 6). However, two isolate IDs are also included for the corned beef and salami samples collected from food premises A. While each pair of isolates from the same samples were considered genetically related (strain 1), they differed by approximately ten alleles, thus just meeting the criteria for relatedness

^**^ “Open food” indicates the product was not intact in its original packaging before testing. “Closed food” indicates the product was intact in the original packaging prior to testing

The CFIA conducted a traceback of the five different *L. monocytogenes*-positive open deli meat samples from food premises A. Two different manufacturing facilities were identified, however the manufacturing facility for one of the *L. monocytogenes*-positive open samples (black forest ham) could not be determined due to a lack of packaging details. The CFIA collected and tested a roast beef sample and a corned beef sample still intact in the original packaging from the same lots of product as the *L. monocytogenes*-positive open samples. Closed samples of the other deli meat products (salami, black forest ham, and the other lot of corned beef) from the same lots as the *L. monocytogenes*-positive open samples were either no longer available or could not be identified due to lack of packaging details. *L. monocytogenes* was not detected in the closed corned beef sample tested, but was detected in the closed roast beef sample. Additional lots of closed roast beef products were also tested and environmental swabs were taken from the associated production line at manufacturing facility Y. *L. monocytogenes* was detected in additional lots of product and on the associated production line (slicer and cooler/snap chiller). As a result of these findings, production on the affected line stopped. Several voluntary product recalls and public warnings were issued, the first of which occurred in late March 2018 and continued until all potentially affected product within shelf life was recalled in mid-April 2018.

Multiple strains of *L. monocytogenes* (both within a sample and across samples) were identified by WGS in the closed roast beef samples and environmental swabs collected at manufacturing facility Y. However, none of the *L. monocytogenes* isolates detected in the closed roast beef samples or environmental swabs from the facility were genetically related by WGS to the open samples from food premises A, outbreak cases, or other confirmed cases of listeriosis. Further, enumeration results indicated very low levels of *Listeria* contamination (< 5 CFU/g) in the closed roast beef samples, which were tested towards the end of their shelf life. After implementing corrective measures, the manufacturing facility resumed operations at the end of April 2018. Food premises A re-opened in late March after all food safety requirements were met, including more frequent slicer cleaning and sanitization (deep cleaning every four hours), staff education on *Listeria*, re-training of staff on slicer cleaning and sanitizing procedures, and 15 *Listeria*-negative environmental swabs (including swabs from the slicer).

### Outbreak two

Towards the end of September 2018 public health officials were notified of a *L. monocytogenes* laboratory-confirmed case who reported consuming a meal during their incubation period at food premises A (Fig. 1). Five days later laboratory results indicated the case had the same first enzyme PFGE pattern as the outbreak-confirmed cases from the first outbreak. Local public health officials collected open food samples and environmental swabs from food premises A. Food premises A was closed following *Listeria* presumptive positive results from the open food samples and environmental swabs. The closure occurred one day after the case was identified as having the same first enzyme PFGE pattern as the first outbreak.

A total of three outbreak-confirmed cases from three different public health units in southern Ontario were included in the investigation. All three outbreak-confirmed cases were considered genetically related by WGS and were also genetically related to outbreak-confirmed cases from the first investigation (within 0 to 10 alleles) (Table 1; Fig. 2). Symptom onset dates ranged from late August to mid-September 2018. All three cases were cancer patients hospitalized as a result of listeriosis. No deaths were reported during the course of the investigation. All three outbreak-confirmed cases reported consuming meals at food premises A during their incubation period. No outbreak-probable cases were identified. Similar to the first investigation, the public health unit in whose jurisdiction food premises A was located issued a public media release and a communication to health care providers. PHO also issued a communication to all public health units in Ontario requesting they share information about the outbreak with health care providers in their jurisdiction.

Local public health officials collected 12 open control food samples and 92 environmental swabs from food premises A. Seven food samples (all various types of deli meat that had been sliced using the on-site slicer) and eight environmental swabs were positive for *Listeria* and were genetically related to the outbreak-confirmed cases by WGS (Table 2). All but one of the *L. monocytogenes*-positive environmental swabs were collected from the slicer – the same slicer that was the suspect source of cross-contamination in the first outbreak. Public health officials permanently removed the slicer from use at food premises A. Food premises A closed due to the investigations.

The same facility as in the previous investigation, manufacturing facility Y, was identified as the *L. monocytogenes*-positive open deli meat manufacturer. As no product labels were available from the *Listeria monocytogenes* positive open product, traceback activities identified what was likely the same lots of product. Closed samples were collected and tested from those lots. *Listeria* was not detected in any of the closed deli meat samples tested. Further, manufacturing facility Y and the CFIA did not report any *Listeria* positive samples during the routine product or environmental testing in the previous three months, which had been scaled up as a result of the first outbreak. No corrective action was required at manufacturing facility Y.

## Discussion

The report describes two *L. monocytogenes* outbreaks that occurred within six months and were linked to the same on-site food premises in an Ontario cancer centre. The centre serves a large in- and out-patient population from across Ontario who visit the centre for medical care. During the outbreaks, the on-site food premises was open to the public and the cancer centre in- and out-patient populations.

Environmental swab results identified the slicer at food premises A as a source of cross-contamination in the second outbreak, and the slicer was the suspected source in the first outbreak. Slicers are known to be a source of growth of *Listeria* that can result in cross-contamination in food premises, as these devices can harbour bacteria (particularly if damaged). Food premises operators must adhere to cleaning and sanitizing requirements to reduce the risk of contamination [39]. Before the first outbreak, proper slicer cleaning and sanitation procedures were not followed. Despite corrective measures, the slicer design did not allow for complete disassembly which likely precluded adequate cleaning and sanitizing. This likely led to the re-emergence of the same genetic strain months later causing the second outbreak. As a result of the two Ontario outbreaks, food premises A in the cancer centre closed.

*Listeria* has been known to persist in the environment for years, particularly on food equipment surfaces that cannot be adequately cleaned and sanitized, leading to foodborne illness outbreaks [40]. Two listeriosis outbreaks in a hospital setting in the United States (US) similarly highlighted the challenge of cleaning and sanitizing inaccessible food contact surfaces, and the environmental persistence of *L. monocytogenes* [33, 34]. These US outbreaks were linked to milkshakes produced in a commercial-grade freezer machine that remained contaminated following the first outbreak, despite repeated cleaning and sanitization, causing a second outbreak of the same strain of *L. monocytogenes* a year later.

The source of the introduction of *Listeria* contamination at food premises A was not determined. Multiple genetic strains of *L. monocytogenes* were identified in closed deli meat samples, and in the environment of the associated production line at manufacturing facility Y. Studies elsewhere have also reported multiple *L. monocytogenes* isolates at production facilities [30, 41]. However, the same genetic outbreak strain found in the laboratory-confirmed cases as well as at food premises A was not identified at manufacturing facility Y. Further, in the first outbreak, closed samples of the *Listeria* positive salami (produced at another manufacturing facility) and black forest ham (where the manufacturing facility could not be determined) could not be obtained to determine if they were contaminated with the outbreak strain.

## Conclusions

Similar to recommendations raised in outbreaks investigations elsewhere, the two outbreak investigations raised a number of considerations for health care facilities and institutions serving individuals with immuno-compromising conditions [33–35]. Health care facilities and institutions should consider the potential health risk of foods available to their patient population when procuring on-site food premises. This may require policies promoting greater collaboration between medical and administrative branches of health care facilities in the procurement process for on-site food premises. During the two Ontario investigations, additional health care facilities and institutions in Ontario were identified with on-site premises (including other locations belonging to the same chain as food premises A) that served foods known to be high-risk for those with immuno-compromising conditions. Excluding high risk foods from being served in settings that care for patients with immuno-compromising conditions may reduce the risk of listeriosis for these patients.

Counselling provided by health care providers to patients at increased risk for listeriosis (those with immuno-compromising conditions, pregnant women and the elderly) regarding the health risks associated with foods that are more likely to be contaminated with *Listeria* is an important preventive measure [31, 35, 36]. However, patients may not necessarily adhere to clinical advice. Out-patients who would not have meals prepared by a medical care centre may be more likely to visit on-site food premises. In the two outbreak investigations described in this paper, a number of the outbreak-confirmed cases were out-patients seeking treatment at the cancer centre. To further reduce the risk to these individuals, it is recommended that high risk foods not be served in settings that care for patients at increased risk for listeriosis. Further, recognizing that those at increased risk for listeriosis consume and purchase high risk foods from many food premises in the community, signage at these premises to alert these individuals of the risk of *Listeria* infection associated with these foods could also be considered. In addition, food handlers at any food premises with an onsite slicer should be appropriately educated on proper cleaning and sanitizing techniques and on the importance of frequent slicer cleaning [42]. Routine public health inspections of food premises should monitor and re-enforce proper cleaning and sanitizing of slicers.

## Data Availability

Case data is closed, and reported in the Ontario Ministry of Health’s integrated Public Health Information System (iPHIS). Public Health Ontario (PHO) cannot disclose the underlying data. Doing so would compromise individual privacy contrary to PHO’s ethical and legal obligations. Restricted access to the data may be available under conditions prescribed by the Ontario *Personal Health Information Protection Act, 2004*, the Ontario *Freedom of Information and Protection of Privacy Act*, the *Tri-Council Policy Statement: Ethical Conduct for Research Involving Humans (TCPS 2 (2018))*, and PHO privacy and ethics policies. Data are available for researchers who meet PHO’s criteria for access to confidential data. Information about PHO’s data access request process is available on-line at https://www.publichealthontario.ca/en/data-and-analysis/using-data/data-requests. The whole genome sequencing datasets generated during and/or analyzed during the current study are available in the National Centre for Biotechnology Information repository within BioProject PRJNA563085, https://www.ncbi.nlm.nih.gov/.

## List of Abbreviations

MOH: Ontario Ministry of Health
iPHIS: Integrated Public Health Information System
PHO: Public Health Ontario
CFIA: Canadian Food Inspection Agency
WGS: Whole genome sequencing
PFGE: Pulsed field gel electrophoresis
NML: National Microbiology Laboratory
